# Psychological distress experienced by nurses amid the fifth wave of the COVID-19 pandemic in Hong Kong: A qualitative study

**DOI:** 10.3389/fpubh.2022.1023302

**Published:** 2023-01-13

**Authors:** Ankie Tan Cheung, Laurie Long Kwan Ho, William Ho Cheung Li, Joyce Oi Kwan Chung, Graeme D. Smith

**Affiliations:** ^1^The Nethersole School of Nursing, Faculty of Medicine, The Chinese University of Hong Kong, Hong Kong, Hong Kong SAR, China; ^2^School of Nursing, The Hong Kong Polytechnic University, Hong Kong, Hong Kong SAR, China; ^3^Caritas Institute of Higher Education, Hong Kong, Hong Kong SAR, China

**Keywords:** COVID-19, nurses, mental health, psychological distress, pandemic

## Abstract

**Introduction:**

The fifth wave of COVID-19 has significantly overburdened the health care system in Hong Kong. Health care professionals, particularly nurses continue to experience significant levels of psychological distress when tackling this ongoing outbreak. Yet, no study has explored the psychological experiences of nurses during the most recent outbreak of the highly transmissible Omicron variant in Hong Kong. The aim of this qualitative study was to explore the psychological distress experienced by nurses during the fifth wave of the COVID-19 pandemic in Hong Kong.

**Methods:**

Twenty-two nurses (14 female and 8 male nurses; average age, 36.7 ± 8.5 years) were recruited to attend the one-to-one semi-structured telephone interviews from June to July, 2022. Data were analyzed using thematic analysis.

**Results:**

Four main themes emerged from the interview: (1) Intense fear, worry, and anxiety; (2) Feeling worn out and psychologically distress; (3) Impact on psychosocial and physical health; and (4) Limited options to cope with psychological distress during the difficult times.

**Discussion:**

Our study findings may provide concerned stakeholders with useful insights into reducing the psychological distress experienced by nurses in Hong Kong. Offering psychological support is of paramount importance to address the unmet psychological needs of nurses and reduce their psychological distress during the pandemic, particularly when they are working under high levels of workplace stress.

## 1. Introduction

The coronavirus disease 2019 (COVID-19) pandemic has spread rapidly across the globe. It has caused a global mental health crisis and continues to stretch the health care system to its limit. As of August 15, 2022, more than 580 million confirmed cases and 6 million deaths have been reported worldwide (1). On November 26, 2021, the World Health organization (WHO) declared the Omicron (B.1.1.529) variant as a global concern based on the advice of the WHO's Technical Advisory Group on SARS-CoV2 Virus Evolution (2). The Omicron variant is the most transmissible among all existing SARS-CoV-2 strains and has become the dominant strain in many countries. Moreover, this highly infectious strain has caused numerous difficulties in the control and management of COVID-19.

Hong Kong's overall COVID-19 tally currently stands at 1,421,918 reported cases, with 9,569 fatalities, as of August 15, 2022 (3). The Omicron variant has caused a fifth wave of the pandemic, pushing Hong Kong's health care system to the brink of collapse. A total of 1,409,287 COVID-19 cases and 9,356 deaths have been recorded since the fifth wave December 31, 2021 (3). Hong Kong's public health care system has been overwhelmed and short-staffed before the pandemic emerged (4). With the unpredictable nature of the COVID-19 pandemic, there is a constantly changing demand for nurses, which has been epitomized by the fifth wave of COVID-19 in Hong Kong. Hong Kong seems to have an effective response to deal with the pandemic by implementing a “zero-tolerance” policy since 2020. However, owing to the highly contagious Omicron variant in Hong Kong in late 2021, there is an unprecedented surge in the number of older and critically ill patients with COVID-19 amid the fifth pandemic wave, further adding burden on the health care system (5). In addition, the entire nursing workforce is currently facing a significant challenge as the fifth COVID-19 wave in Hong Kong has exacerbated the shortage of nurses and other health care professionals. Health care professionals, particularly nurses, are at increased physical and psychological risks due to direct patient care (6–8). A study reported that nurses are experiencing unprecedented psychological distress and burnout amid the pandemic (9). These may be attributed to elevated occupational risks, including occupational infections with COVID-19, limited resources, having to work in understaffed clinical areas, disruption to work-life balance, stigma, and discrimination (10). A meta-analysis of 55 cross-sectional studies involving 189,159 participants belonging to the general population and the health care sector across countries reported that 16.0% of the participants experienced depression symptoms, 15.2% experienced anxiety symptoms, and 22.0% experienced post-traumatic stress symptoms amid the COVID-19 pandemic (11). Of note, a study found that nurses reported more severe symptoms of insomnia and psychological distress than other health care professionals (12). Moreover, frontline jobs may contribute to the risk of poor psychological wellbeing (12, 13) as health care professionals engaged in direct diagnosis, treatment, and care of patients with confirmed/suspected COVID-19 (12).

A few studies have reported health care professionals' experiences of caring for patients with COVID-19 during the early stages of the COVID-19 outbreak (14–17). The fifth wave of COVID-19 has significantly overburdened the health care system. Health care professionals continue to experience significant levels of psychological distress during this ongoing outbreak. However, to the best of our knowledge, no study has explored the psychological experiences of nurses during the most recent outbreak of the highly transmissible Omicron variant in Hong Kong. It is crucial to bridge this gap in the literature to design and provide appropriate interventions to address the psychological needs of nurses in Hong Kong during the continuing global pandemic. Therefore, this study aimed to explore the nurses' psychological experience amid the Omicron wave in Hong Kong.

## 2. Materials and methods

### 2.1. Aim, study design and participants

This exploratory qualitative study was conducted to explore the psychological distress experienced by nurses during the fifth wave of the COVID-19 pandemic in Hong Kong. A research nurse first recruited nurses through word-of-mouth sampling (i.e., passing information regarding this study within the communication network of nurses via a professional nursing organization). This was then followed by the use of a purposeful and snowball sampling technique to recruit registered nurses in different acute hospitals in Hong Kong; i.e., one interviewee provided the name of at least one more potential interviewee for recruitment, and so on. The inclusion criteria were as follows: (i) working as a nurse at a hospital or in other clinical settings; (ii) providing care to patients with confirmed/suspected COVID-19, and (iii) an ability to communicate in Cantonese.

### 2.2. Data collection

To guide each interview, a semi-structured interview guide with key questions and specific probes was developed by an expert panel. This panel included two professors, an assistant professor, and two postdoctoral fellows, all of whom had extensive experience in conducting qualitative research. We referenced the World Health Organization's definition of health, “Health is the state of complete physical, mental and social health, not just the absence of disease or infirmity” to develop our interview guide (18). Moreover, psychological distress is defined as a state of emotional distress caused by exposure to stressful events that pose a threat to an individual's physical or psychological wellbeing (19). In addition, according to a systematic review published in 2021, factors associated with psychological distress in healthcare workers during an infectious disease outbreak include: physical, psychological and social factors, work role and experience, coping styles, and organizational support (20). Therefore, the guide (Supplementary File 1) focused on the following areas: (i) Nurses” general experience when taking care of patients with suspected/confirmed COVID-19; (ii) the impact of COVID-19 on their psychological; (iii) the impact of COVID-19 on their social and physical wellbeing; (iv) the impact of COVID-19 on their nursing role and practice; and (v) strategies employed to cope with the psychological distress. To ensure consistency, all interviews were conducted over the telephone by the co-first authors (ATC and LLKH) experienced in conducting qualitative interviews. ATC and LLKH are both females, registered nurses and have received training in conducting qualitative research during their postgraduate studies. The interviewers did not have any relationship established with the participants prior to study commencement. Eligible nurses were invited to attend the one-to-one semi-structured telephone interviews. Demographic data, including age, sex, marital status, education level, professional position, years of experience as a nurse, and work department, were collected before the start of the interview. Each interview was audio-recorded with the participants' consent. Data collection was conducted until data saturation was reached, indicating that the interviews no longer extracted new information (21).

### 2.3. Ethics considerations

This study involving human participants was reviewed and approved by The Survey and Behavioral Research Ethics Committee of the Chinese University of Hong Kong (reference no: SBRE-21-0773) and was conducted according to the tenets of the Declaration of Helsinki: Ethical Principles for Medical Research Involving Human Subjects. Prior to the study commencement, the purpose and process of the study were explained to all nurses. The nurses provided their written informed consent to participate in this study. The nurses were assured of complete anonymity and confidentiality of their data and received an explanation about their right to withdraw from the study at any point without stating a reason.

### 2.4. Data analysis

The qualitative data were transcribed verbatim and analyzed using thematic analysis (22). First, the two researchers (ATC and LLKH) read the transcripts line-by-line to familiarize themselves with the entire data set. Next, a set of initial codes was generated by systematically collating the data features. Codes with similar meanings were condensed and sorted to potential themes. The initial themes were then further reviewed to ensure internal coherence before finalizing a set of themes and subthemes. Any discrepancies in the coding decisions were resolved by further discussion with another researcher who checked the consistency and coherence of the thematic codes to ensure that an agreement was reached.

### 2.5. Rigor and trustworthiness

To enhance the rigor and trustworthiness of the study, several strategies were employed. First, to minimize investigator bias during the data analysis, all researchers were interviewed by an independent expert to reflect on and report any predispositions that might affect the interpretation and presentation of the findings (23). Second, a reflexive journal was used to document the process and context throughout the research process to increase transparency (24). Third, to ensure the dependability of the findings, discussion among the research team during the data interpretation phase was continued until consensus on themes was reached (25). This study was reported following the Consolidation criteria for reporting qualitative research (COREQ) checklist (26) (Supplementary File 2).

## 3. Results

Twenty-nine nurses were approached, of which 22 (14 female and 8 male nurses; average age, 36.7 ± 8.5 years) nurses agreed to join the study (with a response rate of 75.9%) and were interviewed between June and July 2022. Interviews lasted 35–47 min. Table 1 presents the demographic characteristics and clinical experiences of the participants. Four main themes emerged from the collected data.

**Table 1:** Participants' demographic and work characteristics.

**Characteristics**	***n*** **(%)**
**Age (years)**	
20–29	4 (18.2)
30–39	11 (50.0)
40–49	5 (22.7)
50–59	2 (9.1)
**Sex**	
Male	8 (36.4)
Female	14 (63.6)
**Marital status**	
Single	8 (36.4)
Married	14 (63.6)
**Education attainment**	
Bachelor's degree	7 (31.8)
Master's degree	12 (54.5)
Doctoral degree	3 (13.6)
**Year of working experience**	
1–5	3 (13.6)
6–10	9 (40.9)
11–15	3 (13.6)
>15	7 (31.8)
**Position**	
Registered nurse	13 (59.1)
Advanced practice nurse	7 (31.8)
Ward manager	1 (4.5)
Nurse consultant	1 (4.5)
**Department unit**	
Accident and emergency department	8 (36.4)
Isolation ward	3 (13.6)
Intensive care unit	4 (18.2)
Medical ward	3 (13.6)
Surgical ward	2 (9.1)
Others	2 (9.1)

### 3.1. Theme 1: Intense fear, worry, and anxiety

#### 3.1.1. Subtheme 1.1: Fear of oneself and their family members contracting COVID-19

Many participants expressed their fear of contracting COVID-19 through close contact with hospitalized patients with COVID-19. They mentioned that they were exposed to a contaminated environment containing patients with confirmed COVID-19, potentially infected patients, and their colleagues (who also may have been infected). In particular, some participants were worried that they were at an increased risk of infection when their colleagues tested positive for COVID-19. Most of them felt anxious and feared the associated consequences.

“*As an emergency nurse, I'm exposed to a high-risk environment when providing close direct care for patients. It's inevitable to feel intense anxiety and worry about myself contracting COVID-19, particularly when I realized that my colleagues were confirmed [to be infected]. You know the infection may have multiorgan effects on our body.”* Participant 5.

Most participants expressed their fear of infecting their family members or friends; therefore, they decided to move away from their families to minimize the risk of spreading the infection to their family members and loved ones.

“*I was so worried that I would infect my family members and friends, and I decided to move into a duty room; during that critical moment, I was not at home to see my husband or my kids for nearly one month. It was first at Easter that I went back home as the number of hospitalized patients was gradually decreasing at that time.”* Participant 16.

#### 3.1.2. Subtheme 1.2: Worry and anxiety induced by resource shortages

All the participants highlighted their concerns about resource shortages, including the insufficient availability of personal protective equipment to minimize their exposure to infection at the workplace amid the COVID-19 outbreak This resource shortage posed a threat to their physical and psychological wellbeing.

“*There is inadequate protective gear, including N95 masks and personal protective equipment, to ensure our safety. Imagine you are required to enter airborne infection isolation rooms without adequate protective gear; we were only given surgical masks and face shields to protect ourselves at the time. How can we rest assured that we are safe under such conditions? We are working under threats and worry about the infection risk.”* Participant 4.

Many participants described that there was a limited number of isolation wards and rooms with negative pressure to provide care to suspected/confirmed cases, hence inducing worry and anxiety.

“*The surveillance/isolation wards were not well-established amid the Omicron surge. Due to the high case load, rooms with negative pressure were insufficient to accommodate the confirmed patients. My colleagues and I were all working with anxiety and worry.”* Participant 15.

### 3.2. Theme 2: Feeling worn out and psychologically distressed

#### 3.2.1. Subtheme 2.1: Mental exhaustion and distress due to the increased case load and staff shortage

Many participants described a substantial change in their usual health care operation scenario in response to the rapidly changing pandemic situation in January, with the highly contagious Omicron variant spreading through the community. Many participants experienced immense pressure in response to the evolving demands of COVID-19 in the clinical setting. Owing to the increasing number of older and critically ill patients with COVID-19 during the fifth wave, all of the participants experienced worn out induced by the increased workload during each shift and ever-evolving COVID-19 protocols.

“*I felt worn out as the Omicron outbreak ripped through the city. I work in a surveillance ward, which was responsible for accepting every patient admitted into the hospital for COVID-19 screening and triaging them to other wards after receiving their test results. At the beginning of the fifth wave, many infected people rushed to public hospitals to get treatment as the government advised them to do so. Every staff member was busy: we had to take care of around 16 patients alone, nearly double the number than usual; we could not manage to squeeze in time for lunch/dinner and even had no time for a toilet break throughout our shift.”* Participant 14.

Several participants mentioned that many of their colleagues were infected during the early stages of the Omicron outbreak, causing significant staff shortages. The health care professionals were physically and psychologically exhausted, which in turn resulted in intense distress and mental exhaustion.

“*Due to the surge of the Omicron wave since late February, which was the toughest time we had experienced, there were increasing COVID cases among health care professionals, leading to staff shortages. There were at least 50,000 cases daily in early March, yet at least 10 staff members were being infected in my ward at that moment. In my hospital, there were at least 100 health care professionals infected. Because we originally had barely enough manpower in each shift, losing one or two of them would be very difficult for us. To manage their burden, we must work continuously; everyone was burning out.”* Participant 4.

Some participants described that due to the surge of the fifth wave of the pandemic, there was a rising rate of COVID-19 patients in hospitals, which has resulted in an overall increase in the workload of the health care professionals.

“*I could still remember that nine of the 16 public hospitals had reached full occupancy in late March. I believe that both the health care professionals and patients have been stretched to the limit by the overloading capacity in hospitals with long waiting times. Hundreds of infected older adults in need of treatment and oxygen had no alternative but to stay in the A&E, in corridors, as there were no vacancies in the isolation/surveillance wards.”* Participant 1.

#### 3.2.2. Subtheme 2.2: Feeling worthless, helpless, depressed, and frustrated

Most participants expressed that they felt worthless and helpless when they witnessed many patients with COVID-19 did not receive prompt and appropriate care/treatment during the fifth wave.

“*Frankly speaking, it's like a living hell. When seeing patients lying on the ground, you feel so helpless and worthless to offer care for them. They were like waiting to die, no food, no medicine, and no one was available to offer care for them.”* Participant 2.

Some participants exhibited feelings of depression and frustration when they were unable to provide quality care that met their own high standard to patients with confirmed/suspected COVID-19 due to the high workload.

“*The outbreak was a mess. I felt upset and powerless; after 5 years of being a nurse, I suddenly felt like I knew nothing, I felt unable to help my patients. I broke down and cried, not for myself, but because of the pitiful sight of the patients. That really was the worst experience and most frustrating moment we all had.”* Participant 9.

“*There were many older adults waiting in the cold weather in temporary holding areas outside hospitals, with delayed treatment. It's really unbearable and difficult; we feel like we could be doing more, and I know we can't. I felt like rubbish while working.”* Participant 11.

#### 3.2.3. Subtheme 2.3: Lack of support and low job satisfaction

Some participants revealed that they did not feel understood or supported by their seniors, ward managers, or department heads. They had no opportunity to express their concerns, unmet needs, and expectations to the organization.

“*I would expect our managers to spend time with us in the ward. Even if they do not help, they can just motivate us and support us. But they haven't; we feel lonely.”* Participant 20.

Many participants expressed that there was a lack of support for their wellbeing and workplace safety from the organization amid the fifth wave of COVID-19.

“*Staff's safety and well-being should be the Hospital Authority's top priority. However, we don't feel it. The manager only asked us to wear N95 masks and gowns after the large-scale outbreak in the fifth wave; this never happened in advance as a precaution to protect staff.”* Participant 18.

Some were also concerned about the constantly changing treatment guidelines and policies, which made them feel overwhelmed and unsure as to how to adhere to those guidelines, posing negative impacts on the quality of patient care.

“*We are disappointed with hospital authorities for their slow response and unclear staff instructions, guidelines, and protocols; these could change multiple times, which would lead to confusion among health care professionals. It would also affect patient care.”* Participant 19.

Some participants felt alienated from their profession and expressed that they may quit nursing and reconsider their career path due to low job satisfaction.

“*I no longer wanted to continue in the nursing profession. I mean, I am discouraged by how the profession has been regarded.”* Participant 10.

“*Many colleagues have left during the pandemic: some have emigrated to other countries, while some have moved to private hospitals. I am still making up my mind about whether to resign or not. I had low work satisfaction in the recent half year.”* Participant 4.

### 3.3. Theme 3: Impact on psychosocial and physical health

#### 3.3.1. Subtheme 3.1: Reduced social life/activities

Many participants reported that they tended to stay and work at the hospital continuously and arranged a hotel-based isolation/quarantine during the fifth wave of the pandemic to minimize the risk of infecting their family members and friends.

“*I have had nearly no social life since the fifth wave. I didn't go back home as I know I'm at high risk; I don't want to spread to others, so I chose to stay at a hotel after work.”* Participant 10.

Some revealed that in addition to the social distancing measures imposed by the government, the nature of their work further hampered their social life and activities during the pandemic.

“*I never dated my friends when I was deployed to an isolation ward. I saw that my friends were hanging out with each other on the social media—they were so happy and I'm so lonely. No social life at all.”* Participant 2.

#### 3.3.2. Subtheme 3.2: Perceived stigma of being a nurse and loneliness

Owing to the heightened fear among the public, some participants worried about being stigmatized or discriminated against because of being a health care professional during the COVID-19 pandemic. They perceived some forms of stigma in their daily life from their neighbors, partners, and friends, who maintained an emotional distance from them. All of these factors induced a feeling of loneliness, which in turn jeopardized their psychosocial wellbeing.

“*Some of my friends preferred not to gather during this critical moment, especially those with kids. They were worried about the risk of contracting the virus because of my job. Although I understand their viewpoints, I still feel upset.”* Participant 2

#### 3.3.3. Subtheme 3.3: Muscle pain, migraine, fatigue, stomach pain, and sleep pattern disturbance

Most participants complained about physical discomforts, such as shoulder, neck, and back pain; migraine; and stomach pain after almost every shift. Some reported that these types of discomfort were mainly aftermath of the busy working conditions and the personal protective equipment (e.g., lack of oxygen when wearing N95 masks). Many participants stated that their sleep patterns were disrupted owing to the long shifts and heavy workload and that they always experienced insomnia and nightmares during this unprecedented period. They were craving a break and to take leave, even for a short time, to reset their mind and body.

“*It's an experience I would compare to a world war; I worked like a nurse in the war, encountering busy work and urgent issues in every shift. I was very tired and experienced frequent and severe migraine that usually lasted more than 3 hours; I felt nauseous and stomach pain during migraine. All these impeded my thinking and daily activity.”* Participant 2.

“*There was a tremendous disruption in my sleeping patterns. I always had insomnia and nightmares; my sleep quality is so poor. This has been the greatest impact on me since February.”* Participant 5.

### 3.4. Theme 4: Limited options to cope with psychological distress during the difficult times

#### 3.4.1. Subtheme 4.1: Lack of psychological resources available from organizations

A majority of the participants felt that there was a lack of psychological support from their organizations or that they could not recognize the availability of such support. They mentioned that they felt helpless and that their seniors or supervisors were too busy to give them any support. Some of them revealed that they did not have time to even explore whether any organizational support was available and that they were unable to obtain any related information.

“*I am not sure whether there are any psychological support services from the organization, at least I can't recognize them or have no time to explore.”* Participant 7.

“*We all are very busy, and no one cares about your emotions, even your seniors or supervisors, because they are also occupied by a heavy workload.”* Participant 2.

#### 3.4.2. Subtheme 4.2: Necessity to find ones' own ways to cope

Most of the participants expressed that the stressful working environment made them feel frustrated at times and that it was important for them to find their own ways to cope with the issues. The most reported coping strategies included video or phone calls with family and friends and chats with colleagues. They treasured the time spent with their family and friends, which provided them with a lot of psychological support. In addition to support from family and/or friends, some of them also found other coping strategies that could relieve their psychological distress (e.g., playing phone games, singing, writing diaries, or watching movies).

“*As I work in the dirty team, I need to self-quarantine during or after work to protect my family and friends. Of course, I feel frustrated and lonely sometimes. Luckily, the advanced development of technology allows me to chat with my family and friends via video calls. This gives me the most support for continuing my work.”* Participant 8.

“*I don't know what the proper way to cope with my stress is. Playing phone games is the only way for me to relieve my psychological distress.”* Participant 11.

## 4. Discussion

To the best of our knowledge, this is the first qualitative study that explored the psychological distress faced by nurses in Hong Kong during the fifth COVID-19 pandemic wave caused by the Omicron variant. The pandemic has not only affected public health and the global economy but also caused a remarkable mental health crisis (27–29), in which challenges faced by health care professionals are the greatest. Our findings revealed that nurses are highly vulnerable to COVID-19, given that the highly contagious Omicron variant has not only caused severe and far-reaching repercussions to the health care system but also detrimentally impacted the psychosocial health of nurses. Our findings highlighted that nurses experienced psychological distress amid the COVID-19 pandemic, including feeling worn out, stress, depression, anxiety, worry, fear, and frustration. These findings are consistent with those of previous studies, which imply that the psychological distress experienced by nurses may be largely attributable to the fear associated with the novel coronavirus disease, close personal exposure to infected patients, and elevated stress when caring for patients with suspected/confirmed disease (13, 30). In particular, the findings are supported by a cohort study comprising 158,445 health care professionals and 229,905 of their household members which reported that the risk of hospital admission during the pandemic was 3 times and 2 times higher among the health care professionals and their household members, respectively, as compared with the general population (29). The increased infection risk to health care professionals and the risk of them transmitting the infection to the community could be one of the major factors that caused psychological distress among nurses, as shown in our findings. This pressing issue requires prompt attention from the concerned stakeholders to formulate policies that emphasize the importance of workplace safety and to reduce the occupational risks associated with the health care field and the resultant risk of transmission to health care professionals' families and the community at large.

Insufficient resources in the clinical setting, particularly the number of isolation facilities, availability of personal protective equipment, low work satisfaction, and lack of organizational support, may also be plausible causes of psychological distress among the nurses. Our findings showed that nurses caring for COVID-19 patients may develop fatigue, feeling worn out, anxiety, and mental exhaustion, which corroborates the findings of previous studies (12, 31–33). Another study similarly reported that health care professionals are prone to depression and post-traumatic stress symptoms owing to the COVID-19 pandemic (33). It is noteworthy that 90% of the health care professionals in Hong Kong with a high exposure risk in clinical settings reported mental health problems during the severe acute respiratory syndrome (SARS) outbreak in 2003 (34). Many nurses reported increased levels of anxiety, depression, and post-traumatic stress disorder after the SARS outbreak (35). Of note, some nurses who had been exposed to SARS still required prolonged treatment for their depression and post-traumatic stress symptoms (36). Despite the previous experience of such a large-scale public health crisis in Hong Kong, there is still a lack of resources and psychological support for health care professionals during the COVID-19 pandemic. Nurses still work under immense pressure amid the pandemic which jeopardizes their psychological wellbeing and may lead to compromised quality of care. This reflected that the ingrained problems in the healthcare system in the preparation for public health crises have not been solved since the SARS outbreak.

Offering psychological support is pivotal to addressing the unmet psychological needs of nurses worldwide and reducing their psychological distress during the pandemic, particularly when they are working under high levels of workplace stress. Consistent with findings in other countries (37, 38), our findings also underscore the pressing need for providing psychological support (especially from the organizations/government) for the nurses, as most nurses in this study reported lack of organizational support to cope with the psychological distress during the pandemic. It is worth noting that such organizational support can empower the nurses and hence foster them to cope with the workplace stress more effectively, in additional to self-care and peer support. A multi-faceted approach is warranted to improve and optimize nurses' psychological health (37). This approach may include different components at different times, with strategies for prevention and treatment at various levels, including individual levels by further enhancing the ability of self-care and improving the availability of peer support; organizational levels by offering support or training to enhance the emotional wellbeing and mental health of nurses; and governmental levels by optimizing the health care policies (e.g., improving labor shortage in nurses). All these strategies can prepare nurses, hospitals, and governments for the future possible large-scale public health crisis.

In particular, to provide appropriate psychological support, hospitals should arrange psychological counselors to regularly visit the health care staff and listen to their concerns and stories (39). Meanwhile, it is crucial to enhance the resilience among health care professionals in the face of this public health crisis (40). Resilience, an important psychological construct, is regarded as one's ability to sustain psychological and physical wellbeing in the face of adversity by rebounding from hardship (41, 42). Prior studies have found that building resilience in nurses serves as a protective factor against many mental health problems, such as anxiety and depression, which in turn create beneficial patient outcomes (43, 44). Yet, psychological interventions that aim to foster or enhance psychological resilience in nurses are lacking worldwide (40). Indeed, organizations may provide resilience-promoting interventions for the nurses or other health care professionals to enhance their resilience level and psychological wellbeing amid the pandemic. Appropriate training and resources should also be provided to the nurses for enabling them to access supportive networks (40). For instance, the National Health Service in the United Kingdom offers a national outreach service to nurses for rapid access to mental health support (37). These organizational supports or outreach services could improve the coping ability and promote psychological wellbeing of nurses, especially during such a large-scale public health crisis.

The limitation of this study was the use of the snowball sampling technique to recruit registered nurses from public acute care hospitals, which may result in sampling bias.

## 5. Conclusion

The COVID-19 pandemic has caused widespread disruption of the global health care system and has affected the psychosocial wellbeing of nurses in Hong Kong. Our study findings may provide concerned stakeholders with useful insights into reducing the psychological distress experienced by nurses in Hong Kong. Appropriate psychological interventions should be provided to improve the psychological wellbeing of the health care professionals amid the COVID-19 pandemic.

## Data availability statement

The raw data supporting the conclusions of this article will be made available by the authors, without undue reservation.

## Ethics statement

The studies involving human participants were reviewed and approved by the Survey and Behavioral Research Ethics Committee of the Chinese University of Hong Kong (reference no: SBRE-21-0773). The patients/participants provided their written informed consent to participate in this study.

## Author contributions

AC, LH, and WL conceived and designed this study. AC and LH drafted the manuscript and conducted data analysis, with additional advice regarding analyses contributed by WL, JC, and GS. All authors contributed to the article and approved the submitted version.
