# Supplementary material for: Psychological distress experienced by nurses amid the fifth wave of the COVID-19 pandemic in Hong Kong: A qualitative study
Source: Front Public Health. 2023 Jan 13;10:1023302. doi: 10.3389/fpubh.2022.1023302 (PMC9880411; doi:10.3389/fpubh.2022.1023302)
Supplement: Supplementary file 1 [file Data_Sheet_1.docx]

**Supplementary file 1: Semi-structured Interview Guide**

1. General experience when taking care of patients with suspected/confirmed COVID-19
   1. Can you describe your work environment or experience during the pandemic?
   2. Has anything changed after the pandemic? If yes, please describe more.
2. Impact of COVID-19 on psychological well-being
   1. What was your psychological experience amid the fifth wave of COVID-19 in Hong Kong?
   2. How do you feel about the experiences you mentioned?
   3. How has the pandemic and your work affected your psychological well-being?
   4. How do you interpret the changes related to your psychological well-being caused by the pandemic? Why?
3. Impact of COVID-19 on social and physical well-being
   1. How has the pandemic and your work affected your social life? Why?
   2. How has the pandemic and your work affected your physical well-being? Why?
4. Impact of COVID-19 on nursing role and practice
   1. How do you see your role during the pandemic? Why?
   2. How has the pandemic affected your nursing practice? How do these affect you? Why?
   3. Share the support provided by the organization, if any.
5. Strategies employed to cope with the psychological distress
   1. Have you implemented any strategies to cope with the psychological distress you are experiencing during COVID-19?
   2. If yes, what strategies were used? Please describe more.
